# Highly Efficient Photocatalytic Degradation of Hydrogen Sulfide in the Gas Phase Using Anatase/TiO_2_(B) Nanotubes

**DOI:** 10.1021/acsomega.1c07294

**Published:** 2022-04-01

**Authors:** Yukino Uesugi, Haruki Nagakawa, Morio Nagata

**Affiliations:** Department of Industrial Chemistry, Graduate School of Engineering, Tokyo University of Science, 12-1 Ichigayafunagawara-cho, Shinjuku-ku, Tokyo 162-0826, Japan

## Abstract

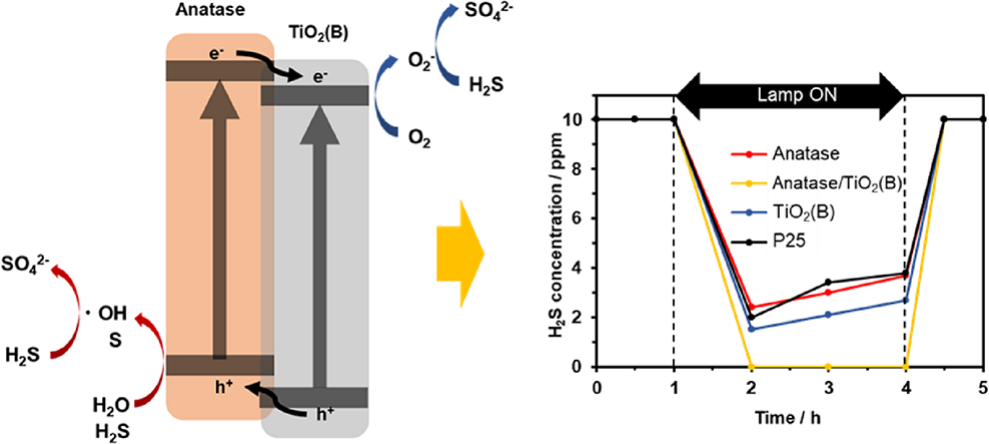

Hydrogen sulfide (H_2_S) is a highly toxic and corrosive gas that causes a foul odor even at very low concentrations [several parts per billion (ppb)]. However, industrially discharged H_2_S has a concentration range of several tens of ppb to several parts per million (ppm), which conventional methods are unable to process. Therefore, advanced and sustainable methods for treating very low concentrations of H_2_S are urgently needed. TiO_2_-based photocatalysts are eco-friendly and have the ability to treat environmental pollutants, such as low-concentration gases, using light energy. However, there are no reports on H_2_S decomposition or oxidation at concentrations below several ppb. Therefore, in this study, we employed anatase/TiO_2_(B) nanotubes, which have a high specific surface area and an efficient charge-transfer interface, to treat H_2_S. We successfully reduced 10 ppm of H_2_S to 1 ppb or less at a kinetic rate of 75 μmol h^–1^ g^–1^. The suitability of our method was further demonstrated by the generation of sulfate ions and sulfur (as detected by X-ray photoelectron spectroscopy and ion chromatography), which are industrially useful as oxidation products, whereas sulfur dioxide, a harmful substance, was not produced. This is the first study to report H_2_S decomposition down to the ppb level, providing meaningful solutions for malodor problems and potential health hazards associated with H_2_S.

## 1 Introduction

In recent years, environmental damage and health problems caused by air pollution have become more serious with rapid economic development. Hydrogen sulfide (H_2_S), a major air pollutant, is a highly toxic and corrosive gas associated with the smell of rotten eggs.^1−3^ H_2_S concentrations of 200–300 ppm cause conjunctivitis and respiratory tract irritation after 1 h of exposure; 100–150 ppm causes coughing, eye irritation, and loss of smell after 2–15 min; 50–100 ppm cause conjunctival irritation; and even 2–20 ppm cause nausea, tearing of the eyes, and headaches.^4,5^ Therefore, the Occupational Safety and Health Administration in the United States sets the emission control concentration of H_2_S to 20 ppm,^6^ while in Japan, the Industrial Safety and Health Law standard for the working environment is below 10 ppm.^7^ As such, H_2_S, widely produced by petroleum, geothermal power, and sewage plants,^4^ is processed to a concentration below the regulation value set by the country and then released into the atmosphere.

The Claus process has been widely used to recover elemental sulfur and water from H_2_S gas. This process involves thermal and catalytic reactions and usually has a H_2_S conversion rate of 96–98% depending on thermodynamic limitations.^8^ Many techniques have been developed to treat the tail gas produced by the Claus process, including catalysis,^9^ scrubbing,^10^ biofiltration,^11^ and adsorption strategies.^12−14^ However, conventional processes have certain disadvantages. Catalytic processes require pH adjustment of the solution as well as high-temperature and high-pressure conditions for the reaction. Scrubbing methods require large amounts of chemical substances, such as magnesium hydroxide, whereas in biological methods, it is necessary to maintain an appropriate temperature for bacteria activity and excess sludge is generated.^9−11^ Therefore, there is a need to develop an eco-friendly and simple process for the treatment of H_2_S to protect human health and the environment.

Furthermore, sulfur compounds such as H_2_S have lower odor thresholds than other malodorous substances, such as nitrogen compounds.^15^ Therefore, low-concentration H_2_S on the order of parts per billion (ppb), which cannot be processed by these technologies, remains malodorous when released into the atmosphere. For example, the Yanaizu-Nishiyama geothermal power station in Fukushima Prefecture, Japan, releases up to 60 ppb of H_2_S,^7^ and the Nesjavellir and Hellisheidi geothermal power plants in Reykjavik, Iceland, produce 153 ppb of H_2_S.^16^ However, as set by the World Health Organization, the concentration at which the odor of H_2_S becomes a nuisance is 4.9 ppb over a 30 min average.^5^ In addition, Collins and Lewis reported that 83% of humans can smell the odor of H_2_S at a concentration of 30 ppb, 50% at a concentration of 8 ppb, and 6% at a concentration of 1 ppb.^17^ The odor threshold of H_2_S in Japan is 0.5 ppb, that in Netherlands is 0.3 ppb,^18^ and that in the United States is 0.5 ppb, as set by the Agency for Toxic Substances and Disease Registry.^5^ As the human sense of smell is extremely sensitive, even if the concentration of the released H_2_S is below the emission control concentration and there is no health hazard, the emission concentration may be considered odorous. Therefore, it is necessary to decompose H_2_S to less than 0.3–1.0 ppb to avoid odor problems.

Photocatalytic decomposition is an efficient technique. In particular, titanium dioxide (TiO_2_) is widely used to remove environmental pollutants because it is highly stable, inexpensive, and nontoxic. TiO_2_ can also generate both oxidizing and reducing species or directly oxidize and/or reduce contaminants adsorbed on its surface under UV light.^19−21^ After Canela et al. reported the first photocatalytic decomposition of H_2_S using TiO_2_,^22^ several studies reported the gas-phase decomposition of high-concentration H_2_S (approximately 30–2000 ppm) using TiO_2_-based photocatalysts.^22−24^ However, only a few studies have determined the efficiency of TiO_2_ photocatalysts for the low concentrations of H_2_S (below the emission control concentration) discharged from factories without processing. In addition, to the best of our knowledge, there are no reports on decomposing H_2_S at the ppb level because of the low surface area of the catalyst and inactivation due to charge recombination.

To elucidate the reaction mechanism of H_2_S decomposition, various studies have identified reaction products, including several types of sulfur oxides. For example, Portela et al. showed that H_2_S is oxidized to sulfate (SO_4_^2–^), which accumulates and deactivates the catalyst.^25,26^ However, Alonso-Tellez et al. suggested that SO_4_^2–^ reacts with photogenerated holes to generate the sulfate radical, resulting in SO_4_^2–^ being completely converted into SO_2_ gas.^27^ Grześkowiak et al. showed that H_2_S is oxidized to elemental sulfur and SO_4_^2–^ under dry conditions.^28^ The experimental conditions in these reports, such as H_2_S concentration, humidity, and light intensity, were different, yielding different reaction products. While these previous studies focused on analyzing reaction products, research on the reaction mechanism by controlling the reaction conditions at low H_2_S concentrations is lacking.^29^

We previously used the Degussa P25 TiO_2_ powder, which is conventionally regarded as highly active TiO_2_, to achieve highly efficient H_2_S decomposition. However, H_2_S at a low concentration of approximately 10 ppm was not completely processed. In addition, silver particles or copper particles deposited on P25 as a cocatalyst were used to decompose low concentrations of H_2_S. Although improved activity was observed, complete treatment was not achieved.^30^ In our previous work, we focused on anatase/TiO_2_(B) nanotubes which were found to be more photocatalytically active than P25 owing to a large surface area and efficient interfacial charge transfer.^31,32^ TiO_2_(B) is a widely studied^33−35^ monoclinic metastable phase that has attracted attention as a photocatalytically active TiO_2_ phase. Therefore, in this study, we applied anatase/TiO_2_(B) nanotubes to the gas-phase decomposition of H_2_S and succeeded in decomposing H_2_S to 1 ppb or less, which are concentrations that do not cause odor problems. We also investigated the effects of oxygen, humidity, and sulfur species on the photocatalyst surface on the reaction mechanism. The results showed that anatase/TiO_2_(B) nanotubes are a useful purification material for air pollutants. This photocatalytic method can be proposed as a new approach for decomposing H_2_S to a level of several ppb, which has not be achieved previously, and solving odor problems caused by H_2_S.

## 2 Results and Discussion

### 2.1 Characterization

**Figure 1:**
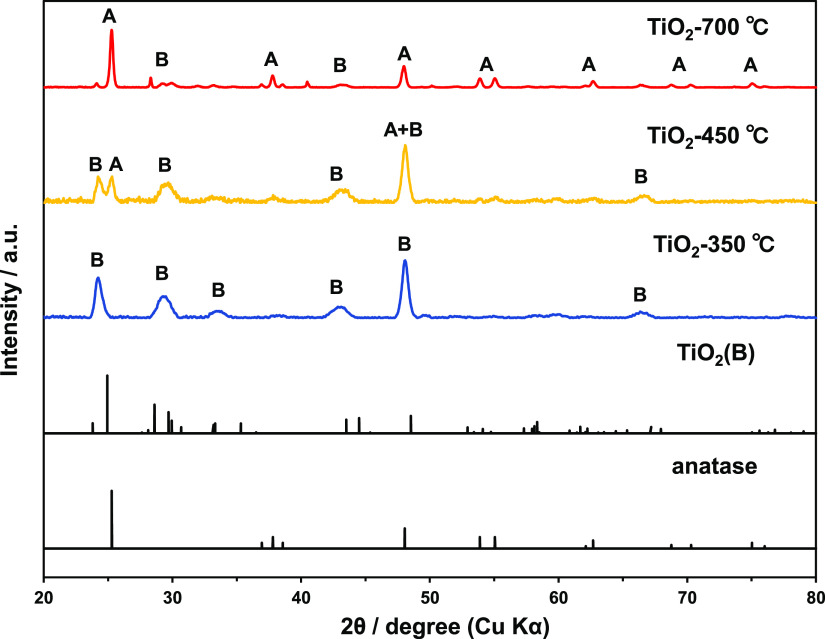
Powder XRD patterns of TiO_2_ calcined at 350, 450, and 700 °C for 2 h and reference patterns for TiO_2_(B) (JCPDS no. 46-1237) and anatase TiO_2_ (JCPDS no. 21-1272).

Figure 1 shows powder X-ray diffraction (XRD) patterns of the TiO_2_-350 °C, TiO_2_-450 °C, and TiO_2_-700 °C samples. The characteristic peaks at 24.2, 29.6, 43.3, 48.5, and 67.2° in the diffraction pattern of TiO_2_-350 °C can be indexed to the (110), (002), (003), (020), and (023) crystal planes of the metastable monoclinic TiO_2_(B) phase (JCPDS no. 00-046-1237), respectively. The main peaks observed for TiO_2_-700 °C at 25.3, 37.8, 48.0, 53.9, 55.1, and 62.7° can be indexed to the (101), (004), (200), (105), (211), and (204) crystal planes of the anatase phase (JCPDS no. 00-021-1272), respectively, while the other peaks at 24.1, 29.3, and 43.3° are attributable to the TiO_2_(B) phase. Based on relative peak intensities, TiO_2_-450 °C shows mixed TiO_2_(B) and anatase phases and TiO_2_-700 °C is mainly composed of anatase, and anatase and TiO_2_(B) are present at a suitable composite ratio in TiO_2_-450 °C. The characteristic anatase peaks intensified as the annealing temperature increased from 450 to 700 °C while those corresponding to TiO_2_(B) decreased. These results indicate that the anatase/TiO_2_(B) composites are formed by the gradual transformation of the TiO_2_(B) phase to the anatase phase, as clarified by the presence of both anatase and TiO_2_(B) in TiO_2_-450 °C.

**Figure 2:**
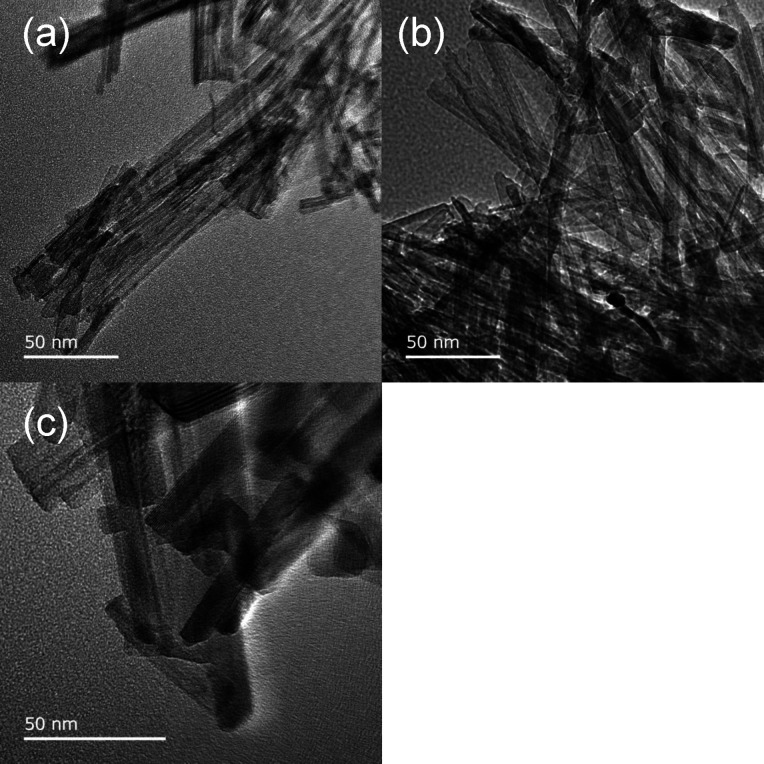
TEM images of (a) TiO_2_-350 °C, (b) TiO_2_-450 °C, and (c) TiO_2_-700 °C.

Transmission electron microscopy (TEM) images of the prepared samples are shown in Figure 2. TiO_2_-350 °C (Figure 2a) has a one-dimensional nanostructure with characteristic nanotubes that are approximately 60 nm long. Increasing the annealing temperature to 450 °C did not result in a considerable morphological change, with the nanotube skeletons being maintained in TiO_2_-450 °C (Figure 2b) owing to the two-step hydrothermal synthesis method.^31^ On the other hand, TiO_2_-700 °C (Figure 2c) has a nanorod structure because the higher annealing temperature destroyed the nanotube structure.

**Figure 3:**
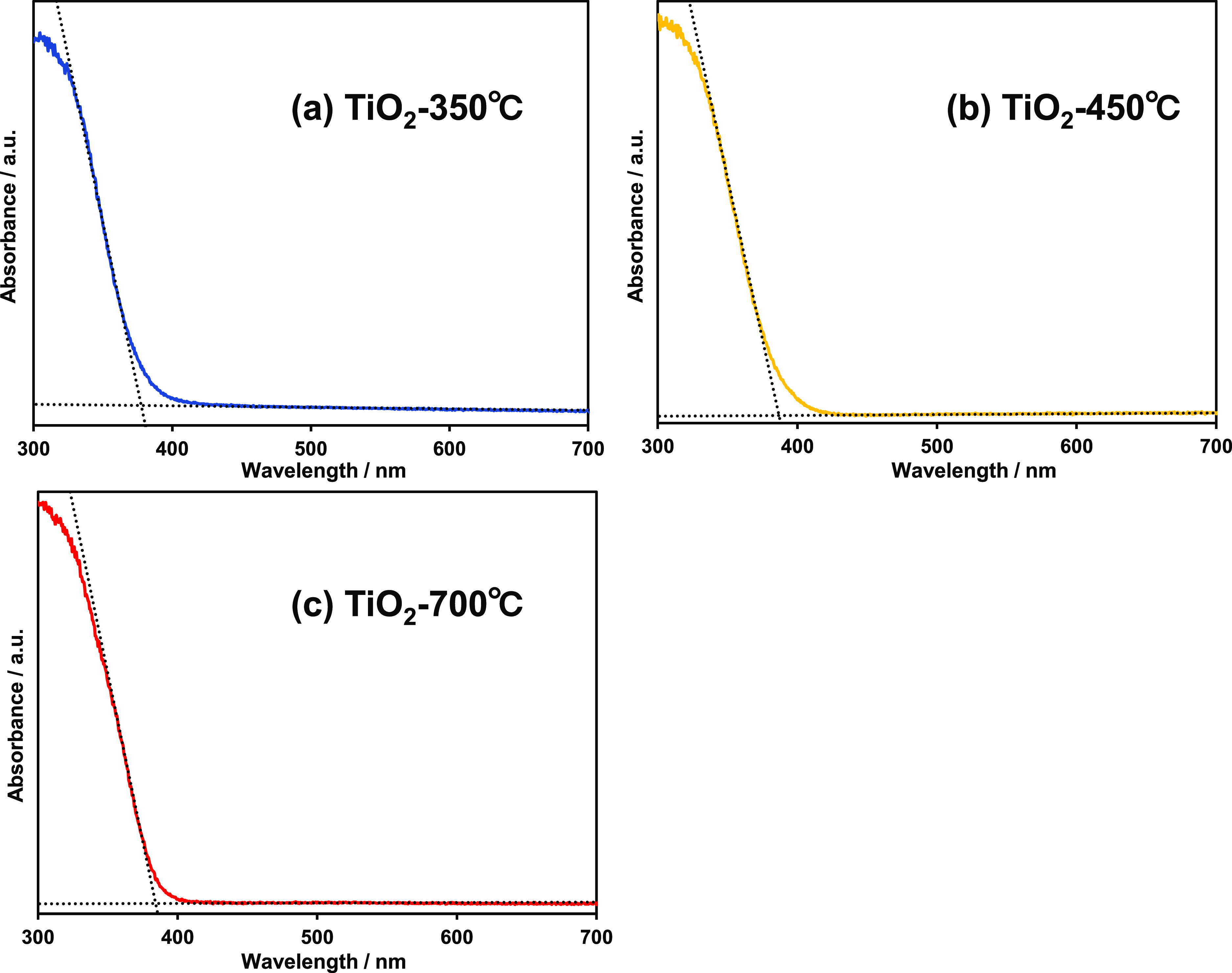
UV–visible diffuse reflectance spectra of (a) TiO_2_-350 °C, (b) TiO_2_-450 °C, and (c) TiO_2_-700 °C.

The diffuse reflectance spectroscopy (DRS) results for the prepared samples are shown in Figure 3. The band gap and absorption edge of each pure material were estimated by extrapolation from the intersection of the slope and the flattened line of the spectra. The calculated band gaps of TiO_2_-350 °C, TiO_2_-450 °C, and TiO_2_-700 °C were 3.29, 3.21, and 3.20 eV, respectively, from which adsorption edges of 377, 387, and 388 nm, respectively, were obtained.

### 2.2 Photocatalytic Activity and Reaction Mechanism

**Figure 4:**
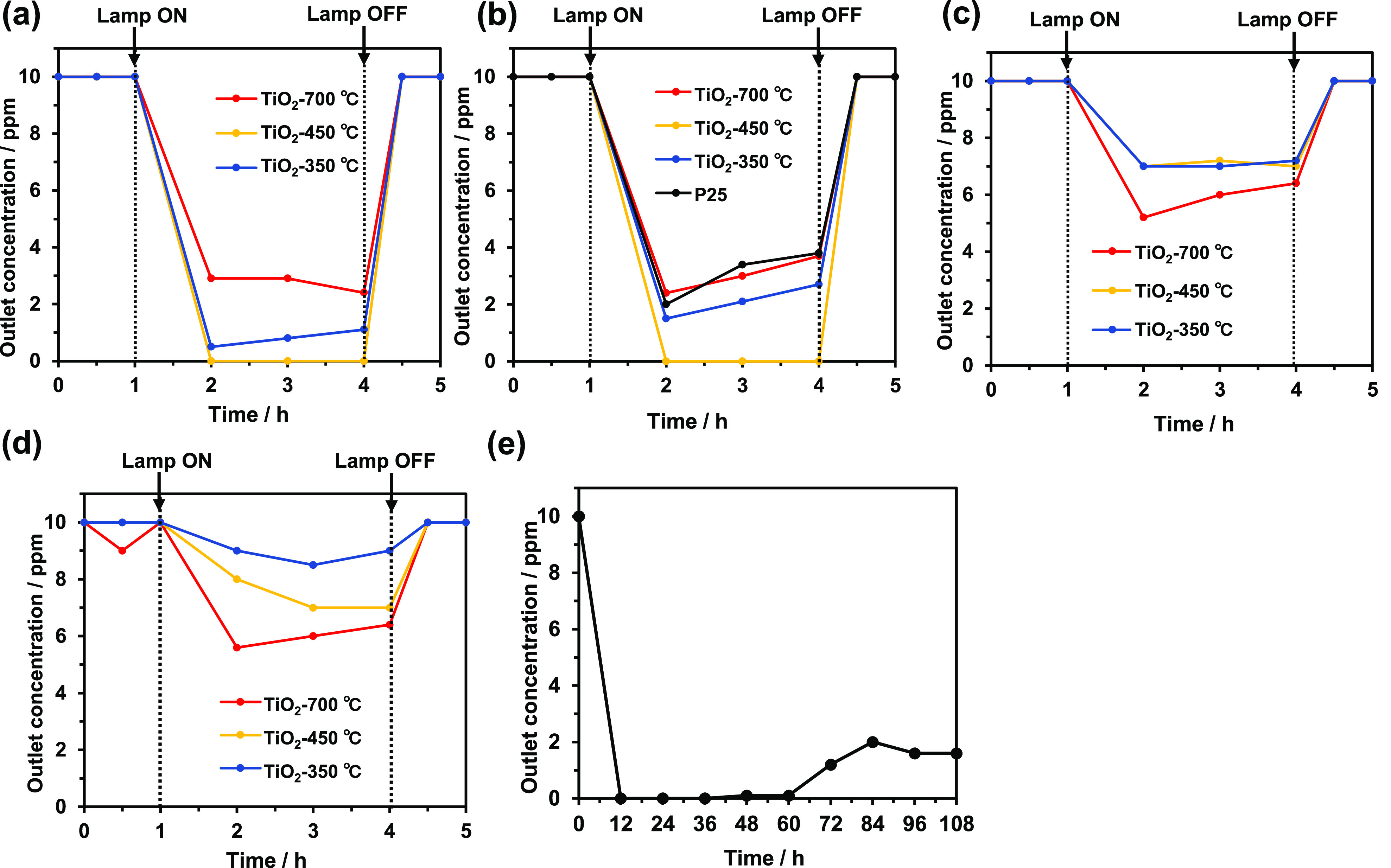
Photocatalytic decomposition of H_2_S with P25, TiO_2_-350 °C, TiO_2_-450 °C, and TiO_2_-700 °C under various conditions: (a) 2–4.6% relative humidity with air as the carrier gas; (b) 18–24% relative humidity with air as the carrier gas; (c) 76–95% relative humidity with air as the carrier gas; (d) 18–24% relative humidity with N_2_ as the carrier gas; and (e) long-term photocatalytic activity during H_2_S decomposition with TiO_2_-450 °C under the same conditions as in (b).

Figure 4b displays the photocatalytic activity during H_2_S decomposition after UV irradiation, with the outlet concentration of H_2_S shown as a function of time. TiO_2_-450 °C exhibited the best performance during gas-phase H_2_S decomposition. Using this photocatalyst, 10 ppm H_2_S was decomposed to 0.1 ppm or less within 3 h. However, the detection limit of the H_2_S concentration detector tube used in this experiment was 0.1 ppm; therefore, it was necessary to determine whether the method yielded H_2_S concentrations at the ppb level. When the measurement was performed using gas chromatography, the H_2_S concentration after 3 h of light irradiation was below the detection limit of gas chromatography (≤1.0 ppb). As 1.0 ppb is the concentration at which 6% of humans detect the odor of H_2_S, the photocatalytic reaction performed in this work can decompose H_2_S to a concentration that is sufficiently low to avoid bad odors. In addition, this is the first report of a ppb level, which is significantly lower than parts per million (ppm) levels previously reported for the photocatalytic decomposition of H_2_S (Table S1).

The superior performance of our method can be attributed to the large surface area and enhanced separation of photogenerated charges induced by the anatase/TiO_2_(B) heterojunction. In our previous work,^32^ the electron energy structure of anatase/TiO_2_(B) nanotubes was suggested using the energy-resolved distribution of electron traps (ERDT) of TiO_2_(B), anatase, and a 1:1 mixture of anatase and TiO_2_(B) measured by reversed double-beam photoacoustic spectroscopy.^36−38^ These results revealed that electron-transfer excitation to the electron traps of TiO_2_(B) occurs based on the high density of states in the valence band of anatase. Furthermore, the valence band top (VBT) of TiO_2_(B) is located at a deeper energy level (approximately 0.7 eV) than the VBT of anatase in the mixture, and the ERDT patterns of the anatase/TiO_2_(B) nanotubes in the mixture match. Using photoluminescence emission spectroscopy and a transient photovoltage technique with a photocatalyst synthesized by the same preparation procedure as in this study, Wang et al. revealed that the electron lifetime of the anatase/TiO_2_(B) composite is longer than that of either anatase or TiO_2_(B).^31^ These results indicate that a type II band structure^39,40^ is constructed between anatase and TiO_2_(B) and that interfacial charge-transfer excitation occurs in the composites. TEM imaging also confirmed the interface between anatase and TiO_2_(B).^31^

**Figure 5:**
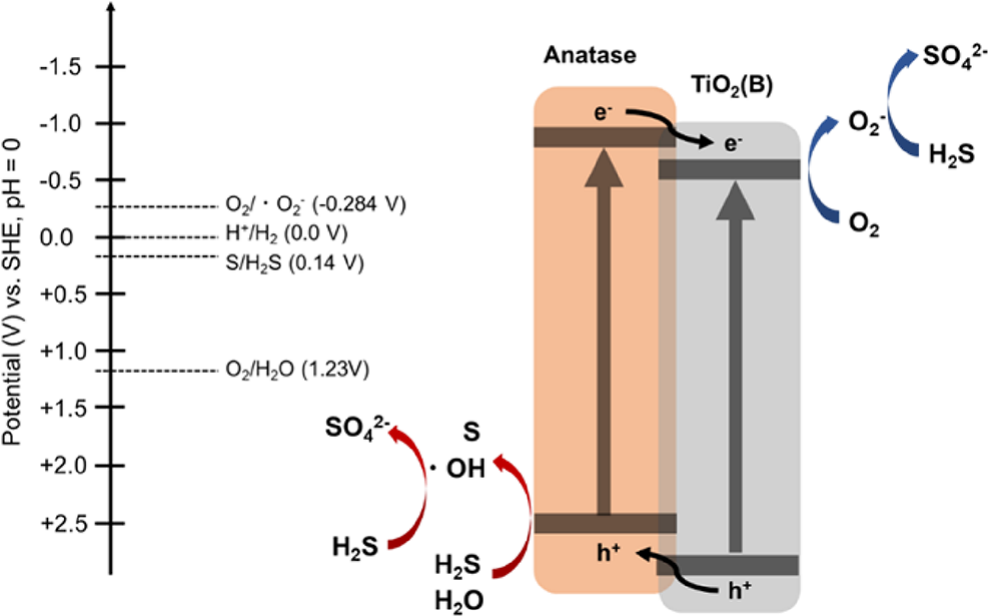
Oxidation mechanism of H_2_S gas using anatase/TiO_2_(B).

Based on these results, we conclude that charge recombination is suppressed by electron transfer in TiO_2_-450 °C (Figure 5). Furthermore, the high photocatalytic activity of TiO_2_-450 °C is due to its larger number of reaction sites and higher specific surface area. Wang et al.^31^ reported that the specific surface area of anatase/TiO_2_(B) nanotubes synthesized by a two-step hydrothermal method is more than 3 times that of P25 or anatase synthesized by two-step hydrothermal methods. The photocatalytic reaction proceeds by adsorbing the decomposed substrate on the surface of the photocatalyst; hence, the specific surface area of the catalyst is important from the viewpoint of adsorption.^41,42^ Therefore, TiO_2_-450 °C, which has a high specific surface area and more reaction sites for H_2_S, exhibits a superior H_2_S-decomposing ability. TiO_2_-350 °C, which has a pure TiO_2_(B) phase, exhibits low photocatalytic activity that is ascribable to charge recombination and the low crystallinity of TiO_2_(B). TiO_2_-700 °C has mixed anatase and TiO_2_(B) phases that promote charge separation; however, it exhibits lower photocatalytic activity owing to the lower specific surface area produced at the higher annealing temperature. The phase compositions, band gaps, specific surface areas, and kinetic rates of TiO_2_-350 °C, TiO_2_-450 °C, and TiO_2_-700 °C are summarized in Table 1.

**Table 1:** Physicochemical Properties and Reaction Kinetic Rates of H_2_S Decomposition of the As-Synthesized Sample

sample	phase composition	band gap [eV]	*S*_BET_ [m^2^ g^–1^]^31^	reaction kinetic rate [μmol g^–1^ h^–1^]
TiO_2_-350 °C	TiO_2_(B)	3.29	303	64
TiO_2_-450 °C	anatase, TiO_2_(B)	3.21	277	75
TiO_2_-700 °C	anatase, TiO_2_(B)	3.20	57	55

**Figure 6:**
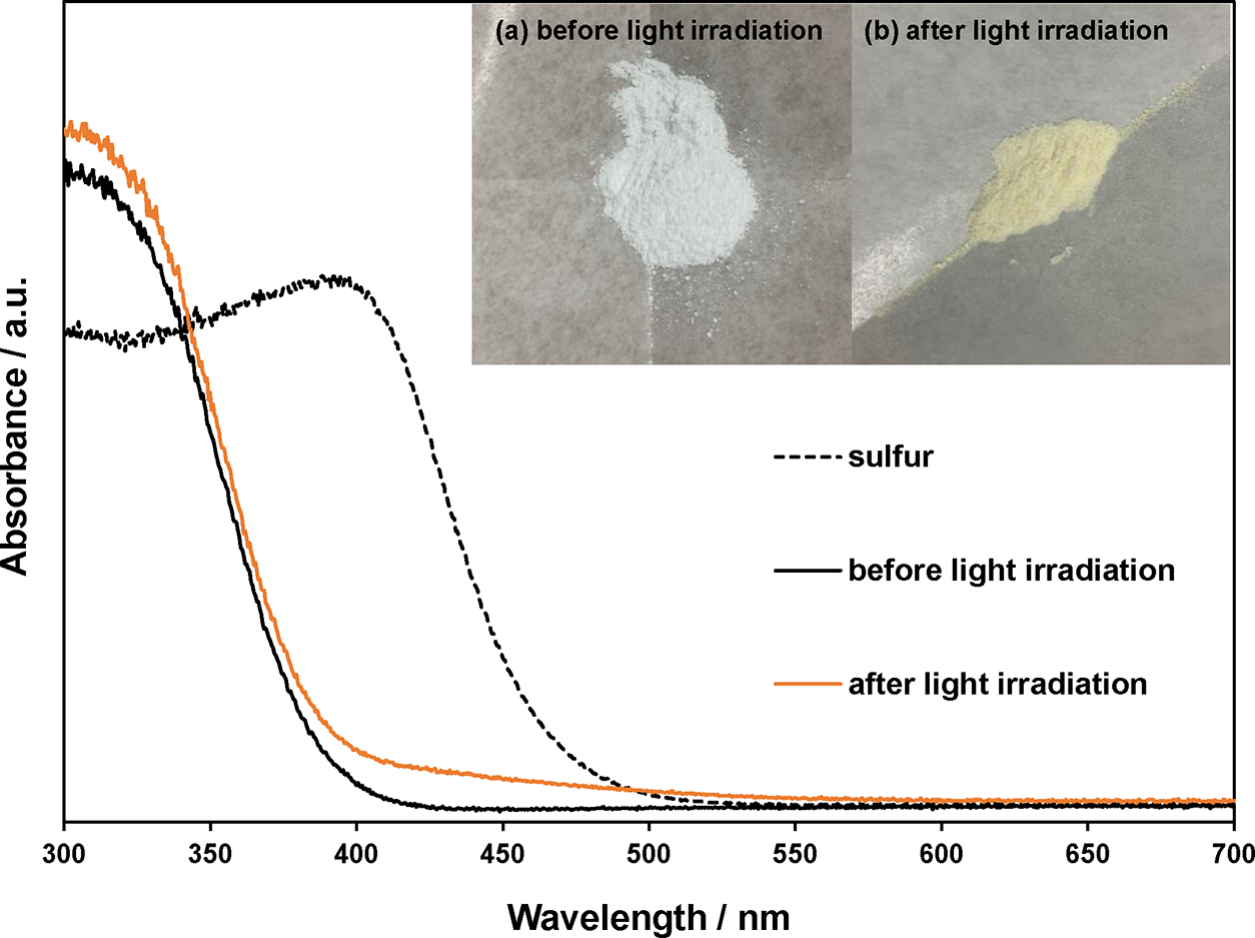
UV–visible diffuse reflectance spectra of TiO_2_-450 °C before and after light irradiation and the color of the photocatalyst (a) before and (b) after light irradiation.

Figure 4d shows the photocatalytic activity for H_2_S decomposition with N_2_ as the carrier gas. Under these conditions, all the photocatalysts decomposed less H_2_S than when air was used as the carrier gas (Figure 4a–c). Therefore, the presence of oxygen is necessary for photocatalytic decomposition. Figure 4c shows H_2_S decomposition under high-humidity conditions (RH = 76–95%), which reveals that the amount of decomposed H_2_S decreases with increasing humidity for all the photocatalysts. Because TiO_2_ is hydrophilic, water molecules are easily adsorbed onto its surface,^20^ which makes it difficult for H_2_S to approach the TiO_2_ surface, thereby inhibiting H_2_S adsorption and decomposition. Under low-humidity conditions (RH = 2–4.6%) (Figure 4a), the amount of decomposed H_2_S by TiO_2_-450 °C was similar to that under normal conditions (RH = 18–24%), whereas TiO_2_-350 °C and TiO_2_-700 °C decomposed more H_2_S under low-humidity conditions than under high-humidity (RH = 76–95%) or normal conditions (RH = 18–24%). Previous reports^22,23^ have mainly considered H_2_S to be decomposed by the hydroxyl radicals generated by oxidizing water molecules with holes. However, in this study, the decomposition reaction proceeded even under low-humidity conditions; therefore, the main reaction is considered to be the direct reaction of H_2_S with holes. We conclude that a H_2_S molecule reacts directly with a hole at the oxidation side and an oxygen molecule reacts with an electron to generate a superoxide anion radical at the reduction side (Figure 5). Since the generated superoxide anion radical has high reactivity, it reacts with H_2_S on the surface of the photocatalyst. In addition, there may be insufficient oxygen or high humidity in environments where H_2_S is actually generated, such as in sewage treatment facilities and geothermal power plants. Figure 4a–d shows that the amount of H_2_S decomposed depends on the reaction conditions. Therefore, selecting reaction conditions suited to the treatment environment is essential when designing a photocatalyst-based H_2_S treatment device. To investigate long-term photocatalytic performance, we used TiO_2_-450 °C as the catalyst under UV light irradiation for 108 h. To replicate use in an actual environment, the photocatalyst was left in the dark for 12 h after irradiation for 12 h, and this experiment was performed for 9 days (Figure 4e). While TiO_2_-450 °C successfully decomposed H_2_S to maintain a concentration of 0.1 ppm or less for 2 d or more, the photocatalytic activity gradually decreased after 3 d of irradiation, with the H_2_S concentration increasing to 2.0 ppm at 84 h. The colors and absorption spectra of TiO_2_-450 °C before and after light irradiation are shown in Figure 6. After light irradiation, TiO_2_-450 °C absorbed at wavelengths of 400–550 nm and changed color from white to yellow.

**Figure 7:**
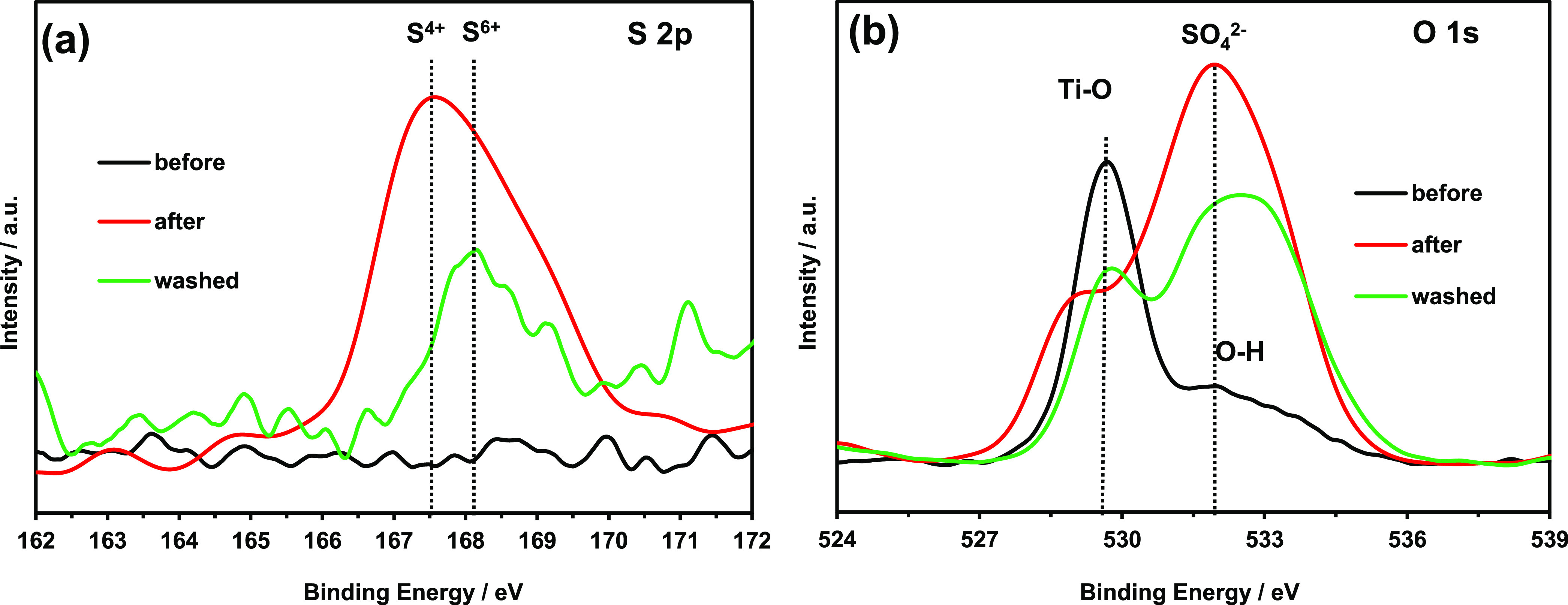
(a) S 2p and (b) O 1s X-ray photoelectron spectra before and after irradiation with light and after photocatalyst washing.

X-ray photoelectron spectroscopy (XPS) was used to investigate the factors affecting the color and photocatalytic activity. The binding energies due to relative surface charging were corrected using the C 1s peak at 285 eV. The S 2p spectra are shown in Figure 7a. A broad peak appeared at 167.6 eV after irradiation with light for 108 h; this peak was further deconvoluted into two peaks at 167.6 and 168.2 eV. The peak at 168.2 eV is due to S^4+^ incorporated into TiO_2_ or oxidized sulfur accumulated on the TiO_2_ surface.^43−46^ The peak at 167.6 eV can be assigned to either the S–O bonds in SO_4_^2–^ or S^6+^ in the TiO_2_ lattice.^45^ The O 1s XPS spectra are shown in Figure 7b. The major peak before light irradiation at 529.7 eV corresponds to O–Ti–O bonds,^47^ and the shoulder peak at 532.2 eV might be due to the O–H bonds of chemisorbed water molecules on the TiO_2_ surface.^48−52^ After light irradiation, the peak at 529.2 eV can be assigned to O–Ti–O and Ti–O–S bonds, and the broad peak at 532.2 eV is due to the S–O bonds in SO_4_^2–^ and the O–H bonds of chemisorbed water molecules,^48−52^ which was expected because SO_4_^2–^ accumulates on the photocatalyst surface during H_2_S decomposition.

To investigate the sulfur species in more detail, TiO_2_-450 °C was washed three times with 5 mL of deionized water after light irradiation, and the washing solution was analyzed using high-performance ion chromatography (HPIC). Only SO_4_^2–^ (7.42 mg) was detected in the washing solution. Because no SO_3_^2–^ was detected, we conclude that the peak at 168.2 eV is not due to S^4+^ in SO_3_^2–^. In addition, the intensities of the peaks at 167.6 and 532.2 eV decreased after washing, suggesting that these peaks correspond to S^6+^ in free SO_4_^2–^ and SO_4_^2–^ adsorbed on the surface of TiO_2_-450 °C. However, a weak peak corresponding to S^4+^ was observed after washing, whereas the peak of elemental sulfur was not detected. Based on these results, we conclude that two factors are responsible for the reduction in photocatalytic activity upon long-term light irradiation. The first is SO_4_^2–^ deposition on the TiO_2_ surface by oxidizing H_2_S to reactive oxygen species and the second is sulfur deposition on the TiO_2_ surface by the direct oxidation of H_2_S with holes (Figure 5). In addition, only the outermost surface of deposited sulfur was oxidized to S^4+^, whereas sulfur at positions deeper than several nanometers from the surface was not oxidized and existed as elemental sulfur. Therefore, the color of the photocatalyst after light irradiation changed to yellow, but only S^4+^ was detected by XPS.

To investigate whether sulfur was doped in the form of S^4+^,^43−45^ TiO_2_-450 °C was washed with hydrochloric acid, and XRD measurements with NaCl added as a crystalline internal standard were performed before and after light irradiation. Figure S1 shows the colors of the irradiated TiO_2_-450 °C before and after washing with 12 M hydrochloric acid. After light irradiation, the catalyst turned yellow, but subsequent washing with hydrochloric acid turned the catalyst white, indicating that sulfur had accumulated on the TiO_2_ surface. Figure S2 shows the XRD patterns of TiO_2_-450 °C before and after light irradiation. No peak shift occurred after irradiation, and no sulfur peak was observed. From these results, it can be concluded that sulfur is not doped in TiO_2_, but a very small amount of sulfur is deposited on the catalyst surface.

To determine the regenerative ability and visible-light responsiveness of the photocatalyst, TiO_2_-450 °C was used for long-term light irradiation, washed with ultrapure water until SO_4_^2–^ disappeared, dried, and reused for H_2_S decomposition under the same experimental conditions as shown in Figure 4b. The amount of decomposed H_2_S did not significantly change in the second cycle compared to the first (Figure S3), and the photocatalytic ability was restored by simply washing with water. When the same experiment was conducted under visible-light irradiation (λ > 420 nm), TiO_2_-450 °C did not decompose H_2_S during the first cycle, whereas approximately 2.0 ppm of H_2_S was decomposed by TiO_2_-450 °C during the second cycle (Figure S4). This behavior implies that sulfur deposited on the TiO_2_ surface functions as a photocatalyst and forms a new band structure with TiO_2_, which exhibits visible-light responsiveness.^53−55^

## 3 Conclusions

In this study, 10 ppm of H_2_S was successfully decomposed to 1.0 ppb or less within 3 h using an anatase/TiO_2_(B) composite photocatalyst owing to its high specific surface area and efficient charge-transfer interface. This study is the first reported example of H_2_S treatment to 1.0 ppb or less using a photocatalyst. We also clarified the effects of changing the reaction conditions, such as the carrier gas and humidity. H_2_S decomposition proceeded best under low-humidity conditions and in the presence of oxygen. Furthermore, XPS and HPIC analyses of the TiO_2_ surface after long-term light irradiation revealed that the reaction mechanism for H_2_S decomposition involved oxygen consumption on the reducing side and holes directly oxidizing H_2_S on the oxidizing side, eventually generating SO_4_^2–^ and sulfur. The photocatalyst after washing was found to decompose 2.0 ppm of H_2_S under visible-light irradiation because sulfur acts as a photocatalyst. In addition, the catalytic activity could be successfully restored by simply washing with water. The findings of this study show that photocatalysis is a useful approach for solving odor problems and provide useful information for designing catalysts to treat H_2_S.

## 4 Experimental Section

### 4.1 Materials

Sodium hydroxide (NaOH, >97%), potassium hydroxide (KOH, >86%), and hydrochloric acid (HCl, 35–37%) were purchased from Kanto Chemical Co., Ltd. TiO_2_ powder (Degussa P25) was obtained from Nippon Aerosil Co., Ltd. All chemicals were used as obtained without further purification.

### 4.2 Photocatalyst Preparation

The TiO_2_ nanotubes were synthesized using a previously reported method.^31^ Degussa P25 (8.46 g) was mixed with 80 mL of 10 M NaOH solution. The mixture was then transferred to a Teflon-lined stainless steel autoclave and heated at 130 °C for 36 h. After this treatment, the autoclave was allowed to cool to 20–25 °C. The intermediate product was then collected and washed several times with deionized water and 0.1 M HCl solution until the pH of the solution was approximately 7. Subsequently, 80 mL of 15 M KOH solution was added. The mixture was transferred to an autoclave, and the temperature was increased to 200 °C for 24 h. The system was cooled to 150 °C at a rate of 1 °C min^–1^ and subsequently maintained at 150 °C for 12 h. The autoclave was then quenched to 20–25 °C. The white product was then collected and washed several times with deionized water and neutralized with the appropriate amount of 0.1 M HCl solution. It was again washed with deionized water to remove any remaining traces of NaCl and KCl. The white product was then filtered and dried at 70 °C for 4 h in air and gently ground in a mortar. Finally, the obtained powder was calcined at 350, 450, and 700 °C for 2 h in air. These samples were labeled as TiO_2_-350 °C, TiO_2_-450 °C, and TiO_2_-700 °C, respectively.

### 4.3 Characterization

The crystal structure of the synthesized TiO_2_ was identified using a powder X-ray diffractometer (Ultima IV, Rigaku) at 40 mA and 40 kV using Cu Kα1 radiation. The morphologies of the TiO_2_ samples were observed using a transmission electron microscope (EM-002BF, JEM-2100, JEOL). DRS was performed using a UV–vis–NIR spectrometer with an integrating sphere (U-3900/3900H spectrometer, Hitachi High-Tech Science). The chemical states of the photocatalysts were investigated using an X-ray photoelectron spectrometer (JPS-9010MC, Mg anode, JEOL). The washing solution was analyzed using a high-performance ion chromatograph (Integrion, Dionex).

### 4.4 Photocatalytic Reaction Setup

**Figure 8:**
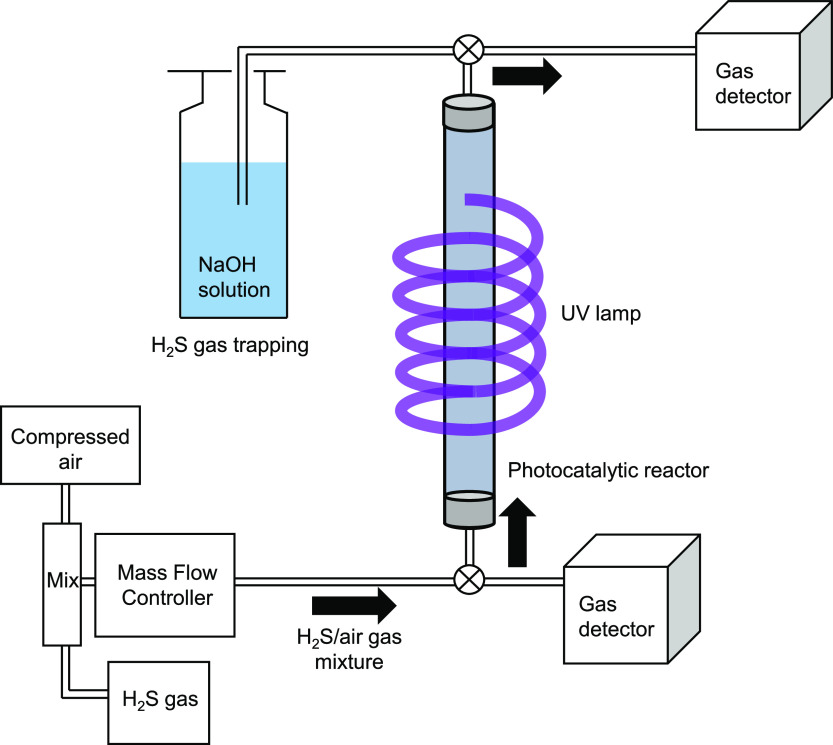
Schematic diagram of the experimental apparatus used to decompose gas-phase H_2_S with a relative humidity of 18–24%. The initial H_2_S concentration was 10 ppm; the flow rate was 0.3 L min^–1^; and the relative humidity was 18–24% under normal conditions, 76–95% under high-humidity conditions, and 2.0–4.6% under low-humidity conditions.

The decomposition of gaseous H_2_S was conducted in a plug flow reactor at room temperature (25 °C). The vessel was made of quartz glass (ID: 17 mm, length: 200 mm). The synthesized photocatalyst (0.1 g) was placed in a glass reaction tube. Compressed air was then circulated to a permeator (PD-1B, GASTEC), followed by heating a permeation tube containing liquid H_2_S (P-4, 380, GASTEC) to 35 °C in the permeator to generate H_2_S gas. A mixture of H_2_S gas (10 ppm) and compressed air (air balance of 300 mL min^–1^) was ultimately produced with a relative humidity of 18–24%; this mixture was then introduced into the glass reaction tube under single-pass conditions. After adsorption equilibration in the dark for 1 h, the reactor was placed below a spiral UV lamp (wavelength: 254 nm, Kyokko Denki Co., Ltd), which emitted UV light with an irradiance of 18 mW cm^–2^. The experimental setup is shown in Figure 8. The decrease in H_2_S concentration was monitored every 60 min for 3 h using a gas detector tube (AP-20-120U, Komyo Rikagaku Kogyo) and a gas chromatograph (GC7100FPD, J-Science Lab Co., Ltd). The flow rate and concentration of H_2_S were adjusted using a permeator. Experiments were conducted under both high- and low-humidity conditions. The high-humidity experiments were performed by bubbling the H_2_S/air gas mixture adjusted with a permeator into deionized water and then circulating this gas, which had a relative humidity of 76–95%, through the reaction vessel. The low-humidity experiments were conducted by passing the gas mixture through a calcium chloride tube, which imparted a relative humidity of 2.0–4.6%, and then passing it through the reaction vessel. The humidity of the generated gas was measured using a digital thermo-hygrometer (SK-120TRH, Sato Keiryoki Mfg. Co., Ltd). To determine the reaction product, the photocatalyst was washed three times with 15 mL of deionized water after light irradiation, and the washing solution was analyzed using HPIC. In addition, the products on the photocatalyst surface after the reaction were analyzed using XPS.

## Supporting Information Available

Comparison of the H_2_S gas decomposition performance of anatase/TiO_2_(B) nanotubes and other photocatalysts; colors of used TiO_2_-450 °C before and after washing with 12 M HCl; XRD patterns of TiO_2_-450 °C before and after light irradiation with NaCl as a crystalline internal standard; photocatalytic decomposition of H_2_S under visible-light irradiation using pure TiO_2_-450 °C and TiO_2_-450 °C that was used and then washed; and photocatalytic decomposition of H_2_S under UV-light irradiation using pure TiO_2_-450 °C and TiO_2_-450 °C that was used and then washed (PDF)

## Abbreviations

XRD: X-ray diffraction
TEM: transmission electron microscopy
DRS: diffuse reflectance spectroscopy
XPS: X-ray photoelectron spectroscopy
HPIC: high-performance ion chromatography
