# Supplementary material for: Highly Efficient Photocatalytic Degradation of Hydrogen Sulfide in the Gas Phase Using Anatase/TiO2(B) Nanotubes
Source: ACS Omega. 2022 Apr 1;7(14):11946–55. doi: 10.1021/acsomega.1c07294 (PMC9016837; doi:10.1021/acsomega.1c07294)
Supplement: Supplementary file 1 — ao1c07294_si_001.pdf [file ao1c07294_si_001.pdf]

Supporting Information

Highly efficient photocatalytic degradation of hydrogen sulfide in the gas phase using anatase/TiO_2(B) nanotubes

*Yukino Uesugi[a], Haruki Nagakawa[a], Morio Nagata[a]**

[†] Department of Industrial Chemistry, Graduate School of Engineering, Tokyo University of Science, 12-1 Ichigayafunagawara-cho, Shinjuku-ku, Tokyo, 162-0826, Japan.

*Corresponding Author: Morio Nagata

E-mails: 4220506@ed.tus.ac.jp (Yukino Uesugi), 4220702@ed.tus.ac.jp (Haruki Nagakawa), nagata@ci.tus.ac.jp (Morio Nagata)

Table of Contents

Table S1. Comparison of various photocatalysts used for decomposing gas-phase hydrogen sulfide (H_2S). S3

Figure S1. Colors of used TiO_2-450 °C (a) before and (b) after washing with 12 M HCl. S4

Figure S2. X-ray diffraction patterns of TiO_2-450 °C before and after light irradiation with NaCl as a crystalline internal standard. S4

Figure S3. Photocatalytic decomposition of H_2S under UV-light irradiation using pure TiO_2-450 °C and TiO_2-450 °C that was used and then washed. S5

Figure S4. Photocatalytic decomposition of H_2S under visible-light irradiation using pure TiO_2-450 °C and TiO_2-450 °C that was used and then washed. S5

Table S1. Comparison of various photocatalysts used for decomposing gas-phase hydrogen sulfide (H_2S).

**Photocatalyst**	**Light source**	**Catalyst [g]**	**Reaction system**	**H_2S concentration [ppm]**	**Reaction kinetic rate [µmol g^{-1} h^{-1}]**	**Ref.**
Anatase/TiO_2(B) nanotubes	UV lamp, λ = 254 nm, 18 mW cm^{-2}	0.1	Flow 0.3 L min^{-1}	Inlet = 10 Outlet => 0.001	>74.86	This work
Degussa P25	30 W black light	3	Flow 0.25 L min^{-1}	Inlet = 250 Outlet = 0.5	51.89	22
TiO_2(anatase)–SiMgOx	8 W UV-A lamp, 4.4 mW cm^{-2}	0.02	Flow 0.075 L min^{-1}	Inlet = 15 Outlet = 2.7	115.1	25
WO_3/TiO_2	8 W black light, 3.3 mW cm^{-2}	0.05	Flow 0.5 L min^{-1}	Inlet = 15 Outlet = 5.85	228.4	27
Ag/TiO_2	UV lamp, 18 mW cm^{-2}, λ = 254 nm	0.2	Flow 0.25 L min^{-1}	Inlet = 12 Outlet = 2.5	29.63	30
Double-shell hollow TiO_2@MIL-101	UV light	0.5	Batch 1.6 L	Inlet = 400 Outlet = 39.6	47.97	23
Anatase film	10 W black light, 1.7 mW cm^{-2}	-	Batch 4 L	Inlet = 50 Outlet = 3	-	24

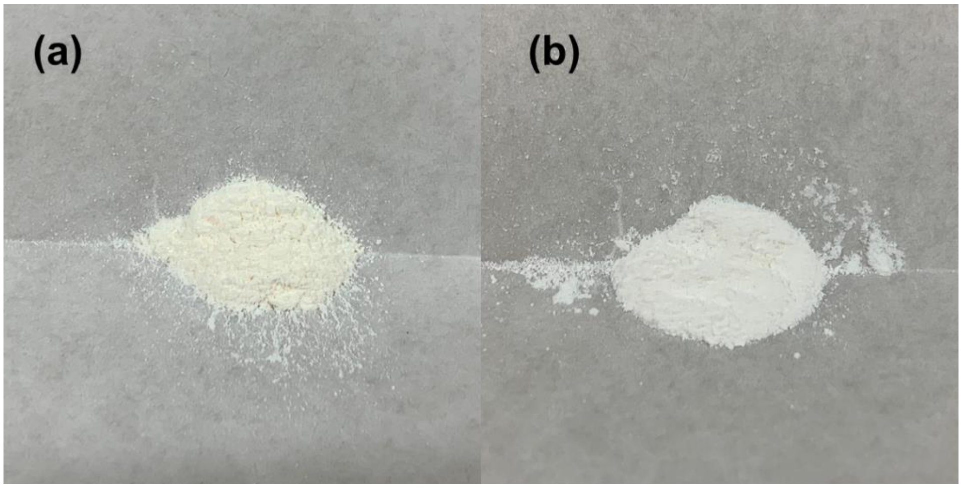

Figure S1. Colors of the used TiO_2-450℃ (a) before and (b) after washing with 12 M HCl.

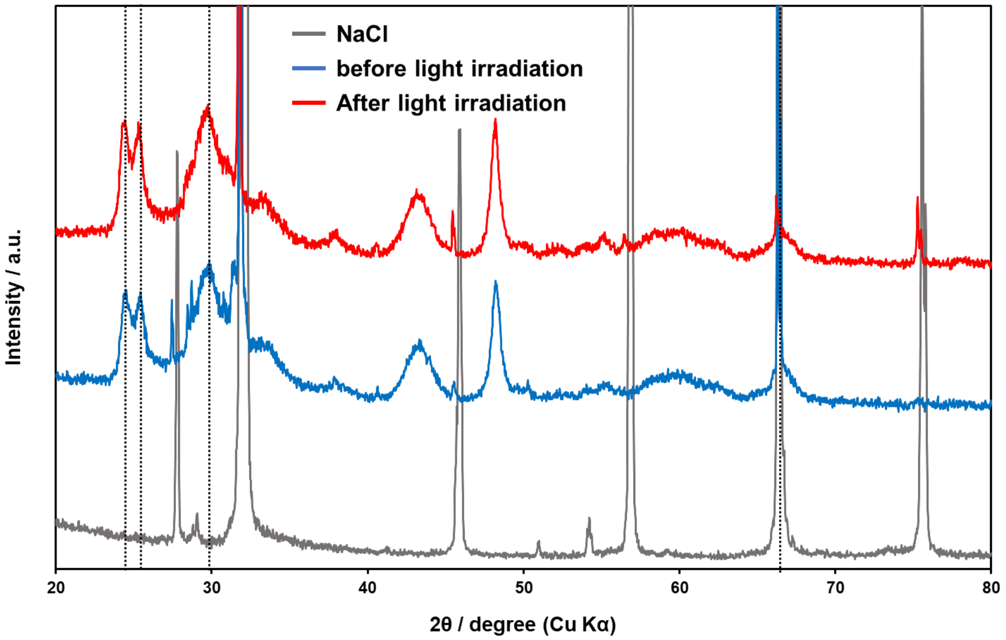

Figure S2. X-ray diffraction patterns of TiO_2-450 °C before and after light irradiation with NaCl as a crystalline internal standard.

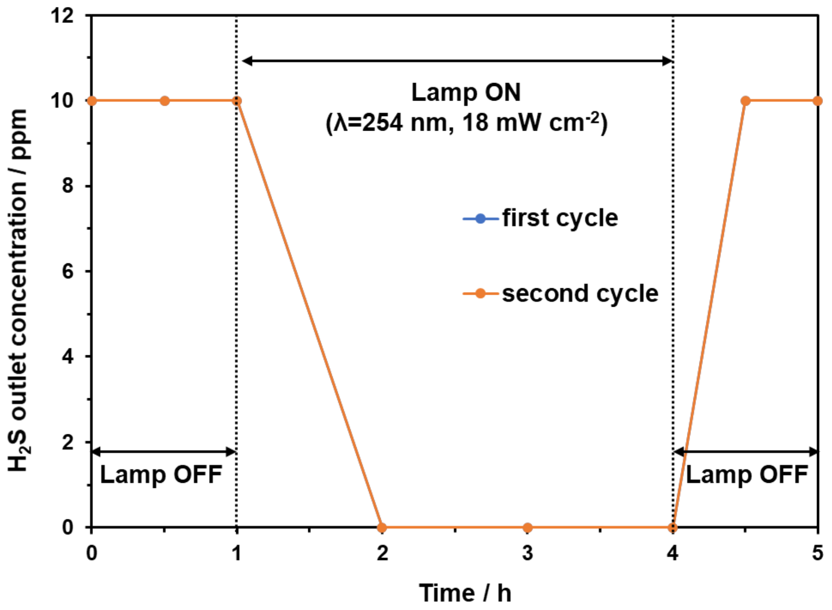

Figure S3. Photocatalytic decomposition of H_2S under UV-light irradiation using pure TiO_2-450 ℃ (first cycle) and TiO_2-450 ℃ that was used and then washed (second cycle).

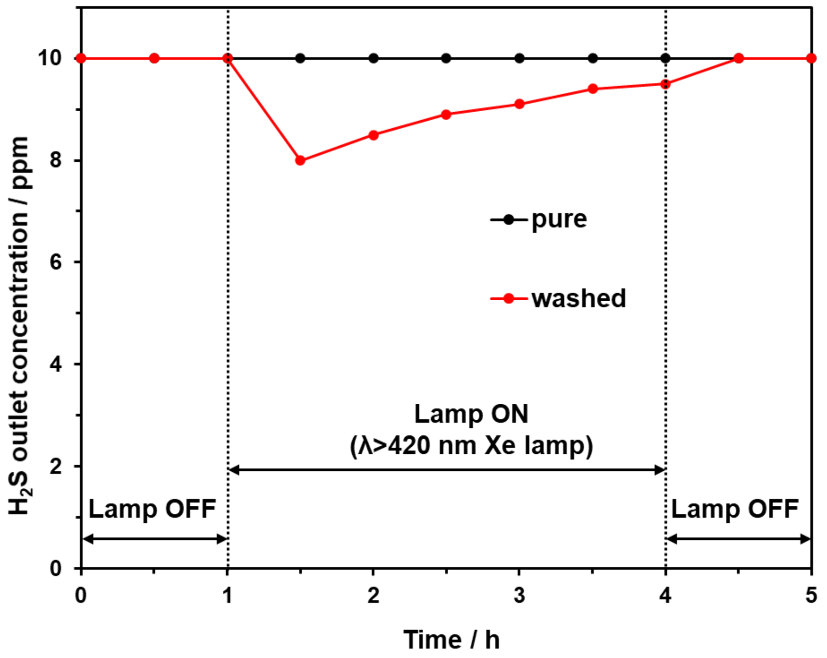

Figure S4. Photocatalytic decomposition of H_2S under visible-light irradiation using pure TiO_2-450 ℃ and TiO_2-450 ℃ that was used and then washed.
